# The effect of smoking on outcomes following primary total hip and knee arthroplasty: a population-based cohort study of 117,024 patients

**DOI:** 10.1080/17453674.2019.1649510

**Published:** 2019-08-02

**Authors:** Gulraj S Matharu, Sofia Mouchti, Sarah Twigg, Antonella Delmestri, David W Murray, Andrew Judge, Hemant G Pandit

**Affiliations:** a Nuffield Department of Orthopaedics, Rheumatology and Musculoskeletal Sciences, University of Oxford, Nuffield Orthopaedic Centre, Oxford;; b Musculoskeletal Research Unit, Translational Health Sciences, Bristol Medical School, University of Bristol;; c Bradford Teaching Hospitals NHS Foundation Trust, St Luke’s Hospital, Bradford;; d Leeds Institute of Rheumatic and Musculoskeletal Medicine, Chapel Allerton Hospital and University of Leeds, Leeds;; e National Institute for Health Research Bristol Biomedical Research Centre (NIHR Bristol BRC), University Hospitals Bristol NHS Foundation Trust, University of Bristol, Southmead Hospital, Bristol, UK

## Abstract

Background and purpose — Smoking is a modifiable risk factor that may adversely affect postoperative outcomes. Healthcare providers are increasingly denying smokers access to total hip and knee arthroplasty (THA and TKA) until they stop smoking. Evidence supporting this is unclear. We assessed the effect of smoking on outcomes following arthroplasty.

Patients and methods — We identified THAs and TKAs from the Clinical Practice Research Datalink, which were linked with datasets from Hospital Episode Statistics and the Office for National Statistics to identify outcomes. The effect of smoking on postoperative outcomes (complications, medications, revision, mortality, patient-reported outcome measures [PROMs]) was assessed using adjusted regression models.

Results — We studied 60,812 THAs and 56,212 TKAs (11% smokers, 33% ex-smokers, 57% non-smokers). Following THA, smokers had an increased risk of lower respiratory tract infection (LRTI) and myocardial infarction compared with non-smokers and ex-smokers. Following TKA, smokers had an increased risk of LRTI compared with non-smokers. Compared with non-smokers (THA relative risk ratio [RRR] = 0.65; 95% CI = 0.61–0.69; TKA RRR = 0.82; CI = 0.78–0.86) and ex-smokers (THR RRR = 0.90; CI = 0.84–0.95), smokers had increased opioid usage 1-year postoperatively. Similar patterns were observed for weak opioids, paracetamol, and gabapentinoids. 1-year mortality rates were higher in smokers compared with non-smokers (THA hazard ratio [HR] = 0.37, CI = 0.29–0.49; TKA HR = 0.52, CI = 0.34–0.81) and ex-smokers (THA HR = 0.53, CI = 0.40–0.70). Long-term revision rates were not increased in smokers. Smokers had improvement in PROMs compared with preoperatively, with no clinically important difference in postoperative PROMs between smokers, non-smokers, and ex-smokers.

Interpretation — Smoking is associated with more medical complications, higher analgesia usage, and increased mortality following arthroplasty. Most adverse outcomes were reduced in ex-smokers, therefore smoking cessation should be encouraged before arthroplasty.

Total hip arthroplasty (THA) and total knee arthroplasty (TKA) are well established clinically and are cost-effective interventions for treating symptomatic arthritis (Learmonth et al. 2007). These procedures are commonly performed worldwide, with numbers predicted to increase (Culliford et al. 2015).

The UK National Health Service is currently under unprecedented financial pressures (Daily Telegraph 2018). In the UK, 197 clinical commissioning groups (CCGs) have the authority and funding to commission healthcare services for their communities. In recent years over half of CCGs have rationed THA and TKA to reduce healthcare expenditure; therefore patients with certain perceived risk factors (like smokers, or those with a high BMI) have been denied access to arthroplasty (Daily Telegraph 2018). A recent report highlighted the severity of the problem with almost 1,700 requests for THA and TKA rejected by CCGs between 2017 and 2018, which represented a 45% increase from the previous year with some CCGs rejecting almost all requests received (Iacobucci 2018a). These actions leave many patients in considerable pain for prolonged periods despite a clinically effective intervention being available, and it appears patients are increasingly accessing arthroplasty in the private sector (Iacobucci 2018a). Thus, the longstanding problem of health inequalities between socioeconomic groups is perpetuated.

Rationing of THA and TKA has been strongly discouraged by surgical bodies (Royal College of Surgeons 2016, British Orthopaedic Association [BOA] 2017). NICE (2017) recommends patient-specific factors (including age, sex, smoking, obesity, and comorbidities) should not be barriers to referral for arthroplasty. Smoking is a modifiable risk factor that is often perceived to adversely affect outcomes following surgery. However, there is insufficient evidence to support the CCGs’ stance of denying current smokers access to arthroplasty. Studies of arthroplasty patients have observed that, compared with non-smokers, smokers have increased wound complications, deep infection, chest infection, implant revision, hospital readmission, and mortality (Singh 2011, Duchman et al. 2015, Singh et al. 2015, Teng et al. 2015, Bohl et al. 2016, Tischler et al. 2017). However, these observations were not consistent between the different studies, with some studies reporting no effect of smoking on these same outcome measures (Inoue et al. 1999, Malik et al. 2004, Sadr Azodi et al. 2008, Musallam et al. 2013, Maoz et al. 2015, Cunningham et al. 2017, Sahota et al. 2018). The inconsistent findings of studies into the effect of smoking on outcomes following arthroplasty might be explained by their numerous limitations including the analysis of small cohorts, not separating THA and TKA patients for analysis, limited or no adjustment for confounding factors, providing only short-term outcomes, and not assessing the effect of previous smoking on outcomes. Furthermore, many studies have not reported on important outcomes, like postoperative analgesia usage and patient-reported outcome measures (PROMs), which have not been assessed in large cohorts. The latter is pertinent given that clinically meaningful improvement in PROMs following arthroplasty is key in deciding whether or not to recommend joint replacement to patients (Wallace et al. 2014). Therefore it is difficult to support the implementation of a policy that denies access to arthroplasty for smokers based on current evidence.

This population-based cohort study assesses the effect of smoking and cessation of smoking on postoperative outcomes following THA and TKA. For completeness we have studied complications, medication usage, hospital readmission, revision surgery, mortality, and PROMs in smokers, ex-smokers, and non-smokers.

## Patients and methods

Patients were initially identified using the Clinical Practice Research Datalink (CPRD) GOLD, which has been described previously (Bayliss et al. 2017). CPRD represents one of the largest databases of longitudinal primary care medical records worldwide. It contains anonymized patient data from 4% of the current UK population (over 2 million patients from 269 contributing practices) (Herrett et al. 2015). Practices’ spread ensures CPRD is representative of the wider UK population for age, sex, and ethnicity. Read Codes are used to enter clinical information (medical history, prescription data, hospital admissions, and interventions), which are standard clinical terminologies used within UK primary care (Benson 2011). CPRD therefore provides a detailed record of both primary and secondary care (Bayliss et al. 2017). The validity and quality of data captured within CPRD have been previously well described (Herrett et al. 2015). A systematic review of validation studies assessing the validity of diagnoses in CPRD identified a large number of studies across a wide range of over 183 different diagnoses and overall estimates of validity were high (Herrett et al. 2010). Aspects of data quality in English primary care are enhanced by the Quality and Outcomes Framework, an incentive payment program for primary care physicians, which encourages recording of key data items (for example smoking status).

Where available, primary care records from CPRD were linked to secondary care admission records from Hospital Episodes Statistics Admitted Patient Care data (HES) and to the Office for National Statistics (ONS) database. HES uses International Classification of Diseases 10th revision (ICD-10) records diagnoses and the Office of Population Censuses and Surveys version 4 (OPCS-4) procedures to record diseases, complications, interventions, and procedures from secondary care. From April 1, 2009, HES provided PROMs data before and 6 months following THA and TKA (see below). The ONS provides data on all-cause mortality.

### Population

All patients aged 18 years and older in CPRD with a diagnostic code for primary THA or TKA between January 1, 1995 and January 28, 2017 were identified using previously validated Read Codes (Culliford et al. 2012, 2015). Patients were eligible for inclusion if their record was labelled acceptable by CPRD quality control (Herrett et al. 2015), approved for CPRD, HES, ONS linkage, and if the patient was registered with their general practice for at least 12 months (n = 136,410). Patients were excluded if data on the exposure variable were missing (n = 19,386) leaving 117,024 patients for analysis (Appendix 1). The study exposure, covariates, and outcomes were identified from the various linked databases using ICD-10 codes, OPCS-4 operation codes, Product Code lists for prescribed medications and Read Codes.

### Exposure

The study exposure was patient smoking status as classified in CPRD at the time of arthroplasty: current smoker, ex-smoker, and non-smoker. Studies specifically assessing the quality of smoking data within CPRD demonstrate prevalence estimates for current smoking and non-smoking that are similar to those from nationally representative surveys, although former smoking may be under-recorded (Booth et al. 2013).

### Covariates

CPRD contains numerous patient-related covariates, which were subsequently adjusted for. These included age, sex, BMI, socioeconomic status, alcohol consumption, year of surgery, and pre-existing comorbidities (including cardiovascular, respiratory, and cerebrovascular diseases, renal failure, cancer, inflammatory arthritis, diabetes). BMI was categorized as underweight (≥10 and <18.5); normal (≥18.5 and <25 ); overweight (≥25 and <30 ); obese class I (≥30 and <35 ); obese class II (≥35 and <40 ); and obese class III (≥40 and ≤60 ). Socioeconomic status was classified using the Index of Multiple Deprivation (IMD), as described previously (Conrad et al. 2018). Patients were divided into 10 equal groups ranked from 1 (least deprived area) to 10 (most deprived area) (Department for Communities and Local Government [DCLG] 2015). The Charlson Comorbidity Index was calculated for each patient based on pre-existing comorbidities. Preoperative PROMs were available for a subgroup of patients (detailed below).

### Outcomes

Postoperative outcomes of interest were: complications, mortality, medication usage, hospital readmission, revision surgery, and PROMs. All outcome data were collected using a combination of CPRD and HES apart from mortality, which was obtained from CPRD and ONS. Complications were recorded within 6 months of surgery, and included cerebrovascular events, myocardial infarction, ischemic heart disease, deep vein thrombosis, pulmonary embolism, lower respiratory tract infection (LRTI), urinary tract infection, and wound infection. Medications prescribed (which included analgesia requirements) and hospital readmissions within 1 year of surgery were recorded. Revision surgery was defined as removal or exchange or addition of any implant(s), within 20 years of surgery. Mortality was assessed 1 year postoperatively. PROMs at 6 months following surgery were available in a subgroup of patients, which are collected as part of a national PROMs program. These joint-specific (Oxford Hip Score [OHS] and Oxford Knee Score [OKS]) PROMs are validated measures for assessing outcomes following arthroplasty. They are scored from 0 (worst) to 48 (best). The change in score following arthroplasty was calculated by subtracting the preoperative score from the 6-month postoperative score. The minimally important clinical differences are 5 points for the OHS and 4 points for the OKS (Beard et al. 2015).

The validity of coding of complications has been previously assessed and known to be good (Wallace et al. 2014). Mortality rates within CPRD are comparable to rates in the National Joint Registry (NJR) annual reports (NJR 2018). Data on revision and readmission come from linked data from HES records, and the validity of coding between NJR and HES records has previously been described in NJR annual reports and data quality audits (NJR 2018). Furthermore validation studies of joint replacement records in CPRD and HES showed high levels of agreement (Hawley et al. 2016).

### Statistics

We assessed the effect of smoking status on binary outcomes (complications, medication usage, and readmissions) by fitting a generalized linear model with a binomial error structure and a log link function (log-logistic model), where results are presented as relative risk ratios (RRR). Models were adjusted for potential confounding factors (age, sex, BMI, IMD, alcohol consumption, year of surgery, and the Charlson Comorbidity Index). Cumulative implant and patient survival rates following arthroplasty were determined using Kaplan–Meier estimates. Patients who were alive with an arthroplasty not requiring revision surgery were censored on the study end date. The associations between smoking status with implant and patient survival rates were explored using Cox regression analysis, with models adjusted for confounders (see above). The confounders we adjusted for were selected a priori given that they have been shown to affect the study exposure, outcomes, and/or access to healthcare (Hunt et al. 2013, 2014, Wallace et al. 2014, Kunutsor et al. 2016, AOANJRR 2018, Edwards et al. 2018). Proportional hazards assumptions were assessed and satisfied for all regression analyses. We used an ANCOVA linear regression model to look at predictors of the obtained 6-month OHS and OKS, adjusting for the baseline score. To look at changes in scores between baseline and follow-up we fitted a repeated measures regression model where the outcome was expanded to include the preoperative and 6-month postoperative OHS or OKS. Interaction terms were fitted between the predictor variable and time, to describe the change in OHS and OKS over time across categories of the predictor variable smoking. We used robust standard errors with the sandwich variance estimator given there was evidence of heteroscedasticity. All models were based on complete case analysis. All statistical analyses were performed with Stata Statistical Software release version IC 15.0, 2017 (StataCorp, Stata College Station, TX, USA).

### Ethics, funding, and potential conflicts of interest

Ethical approval was not required for this study. The Clinical Practice Research Datalink (CPRD) Group has obtained ethical approval from a National Research Ethics Service Committee for all purely observational research with anonymized CPRD data (i.e., studies that do not include patient involvement). The study was approved by the Independent Scientific Advisory Committee (ISAC) for MHRA Database Research, protocol number 17_104R.

Funding was received for this study from departmental funds held at the University of Leeds, England. AJ is supported by the NIHR Biomedical Research Centre at the University Hospitals Bristol NHS Foundation Trust and the University of Bristol.

GSM has received financial support for other research work from Arthritis Research UK, the Orthopaedics Trust, Royal College of Surgeons of England, and Royal Orthopaedic Hospital Hip Research and Education Charitable Fund. GSM has also received personal fees for undertaking medicolegal work for Leigh Day. SM, ST, and AD have no relevant conflicts of interest. AJ has received consultancy fees from Freshfields Bruckhaus Deringer, and is a paid member of the data safety and monitoring board for Anthera Pharmaceuticals. DWM receives royalties from Zimmer Biomet. DWM and HGP are paid consultants for Zimmer Biomet, and both receive institutional research funding from Zimmer Biomet. HGP is also a paid consultant for Kennedys Law, Bristol Myers Squibb, Depuy Synthes, Medacta Int and Meril Life. He has received institutional research grants from UKIERI, Charnley Trust, Depuy Synthes, Glaxo Smith Kline and NIHR. 

## Results

We studied 117,024 patients undergoing arthroplasty (60,812 THAs and 56,212 TKAs) with details available on smoking status (12,644 (11%) smokers, 38,148 (33%) ex-smokers, and 66,232 (57%) non-smokers) (Tables 1 and 2).

**Table 1.** Demographics of patients undergoing total hip arthroplasty. Values are frequency (%) unless otherwise stated

Confounder	Smoker	Non-smoker	Ex-smoker
Total	7,543 (12)	34,271 (56)	18,998 (31)
Male	3,059 (41)	11,343 (33)	9,343 (49)
Female	4,484 (59)	22,928 (67)	9,655 (51)
Age, mean (SD)	63 (12)	70 (11)	70 (10)
BMI			
Underweight	228 (3.7)	526 (1.8)	227 (1.4)
Normal	2,118 (34)	8,692 (30)	3,913 (23)
Overweight	2,217 (36)	11,771 (40)	6,866 (41)
Obese class I	1,140 (19)	5,852 (20)	4,015 (24)
Obese class II	365 (5.9)	1,849 (6.3)	1,356 (8.1)
Obese class III	110 (1.8)	607 (2.1)	392 (2.3)
Missing	1,365 (	4,974 (	2,229 (
Charlson score 1 year a			
0	6,962 (92)	31,437 (92)	16,874 (89)
1	325 (4.3)	1,398 (4.1)	1,052 (5.5)
2	206 (2.7)	1,148 (3.3)	803 (4.2)
≥ 3	50 (0.7)	288 (0.8)	269 (1.4)
Deprivation Index rank			
1	416 (9.6)	3,446 (17)	1,574 (14)
2	418 (9.7)	2,862 (14)	1,453 (13)
3	436 (10)	2,697 (13)	1,444 (13)
4	469 (11)	2,552 (12)	1,387 (12)
5	488 (11)	2,590 (13)	1,480 (13)
6	418 (9.7)	1,908 (9.2)	1,092 (9.4)
7	389 (9.0)	1,635 (7.9)	995 (8.6)
8	402 (9.3)	1,283 (6.2)	882 (7.6)
9	426 (9.9)	959 (4.6)	675 (5.8)
10	461 (11)	756 (3.7)	599 (5.2)
Missing	3,220 (	13,583 (	7,417 (
Alcohol consumption			
Yes	4,691 (80)	21,151 (78)	13,029 (84)
No	898 (15)	5,628 (21)	1,854 (12)
Ex	243 (4.2)	530 (1.9)	555 (3.6)
Missing	1,711 (	6,962 (	3,560 (
Year of surgery			
1995–2000	738 (9.8)	2,980 (8.7)	736 (3.9)
2001–2010	4,291 (57)	18,864 (55)	10,710 (56)
2011–2016	2,514 (33)	12,427 (36)	7,552 (40)
Preoperative Oxford Hip Score			
median (IQR)	16 (11–21)	18 (13–24)	18 (12–24)

a Charlson index score based on comorbidities 1 year prior to index date.

**Table 2.** Demographics of patients undergoing total knee arthroplasty. Values are frequency (%) unless otherwise stated

Confounder	Smoker	Non-smoker	Ex-smoker	
Total	5,101 (9.1)	31,961 (57)	19,150 (34)	
Male	2,597 (51)	10,910 (34)	10,997 (57)	
Female	2,504 (49)	21,051 (66)	8,153 (43)	
Age, mean (SD)	64 (10)	70 (10)	70 (9)	
BMI	Underweight	44 (1.0)	138 (0.5)	63 (0.4)
Normal	844 (20)	4,811 (17)	2,300 (13)	
Overweight	1,628 (38)	10,567 (38)	6,696 (39)	
Obese class I	1,144 (26)	7,744 (28)	5,179 (30)	
Obese class II	488 (11)	3,526 (13)	2,162 (13)	
Obese class III	178 (4.1)	1,390 (4.9)	855 (5.0)	
Missing	775 (	3,785 (	1,895 (	
Charlson score 1 year a	0	4,646 (91)	28,994 (91)	16,985 (89)
1	249 (4.9)	1,451 (4.5)	1,062 (5.5)	
2	161 (3.2)	1,190 (3.7)	857 (4.5)	
≥ 3	45 (0.9)	326 (1.0)	246 (1.3)	
Deprivation Index rank	1	253 (8.2)	2,932 (15)	1,496 (13)
2	272 (8.8)	2,396 (12)	1,407 (12)	
3	276 (8.9)	2,427 (12)	1,459 (12)	
4	310 (10)	2,455 (13)	1,431 (12)	
5	358 (12)	2,363 (12)	1,451 (12)	
6	312 (10)	1,907 (9.7)	1,208 (10)	
7	320 (10)	1,709 (8.7)	1,033 (8.7)	
8	328 (11)	1,445 (7.3)	982 (8.2)	
9	310 (10)	1,092 (5.5)	764 (6.4)	
10	357 (12)	964 (4.9)	705 (5.9)	
Missing	2,005 (	12,271 (	7,214 (	Alcohol consumption
Yes	3,129 (79)	19,436 (75)	13,348 (85)	
No	660 (17)	5,809 (23)	1,859 (12)	
Ex	173 (4.4)	550 (2.1)	570 (3.6)	
Missing	1,139 (	6,166 (	3,373	
Year of surgery	1995–2000	357 (7.0)	1,917 (6.0)	524 (2.7)
2001–2010	3,074 (60)	18,115 (57)	11,101 (58)	
2011–2016	1,670 (33)	11,929 (37)	7,525 (39)	
Preoperative Oxford Knee Score				
median (IQR)	17 (12–23)	19 (14–25)	20 (14–25)	

a Charlson index score based on comorbidities 1 year prior to index date.

### Complications

**Figure 1.**
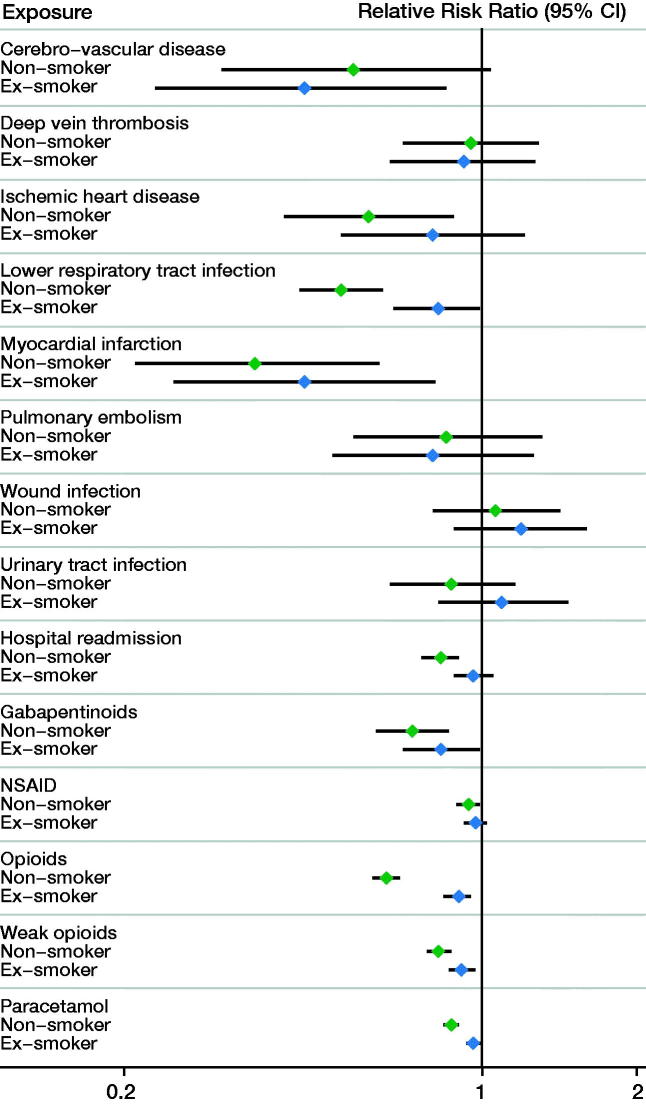
Forest plot for complications and medication usage following total hip arthroplasty by smoking status. The respective relative risk ratios and 95% confidence intervals are provided in Appendix 3.

**Figure 2.**
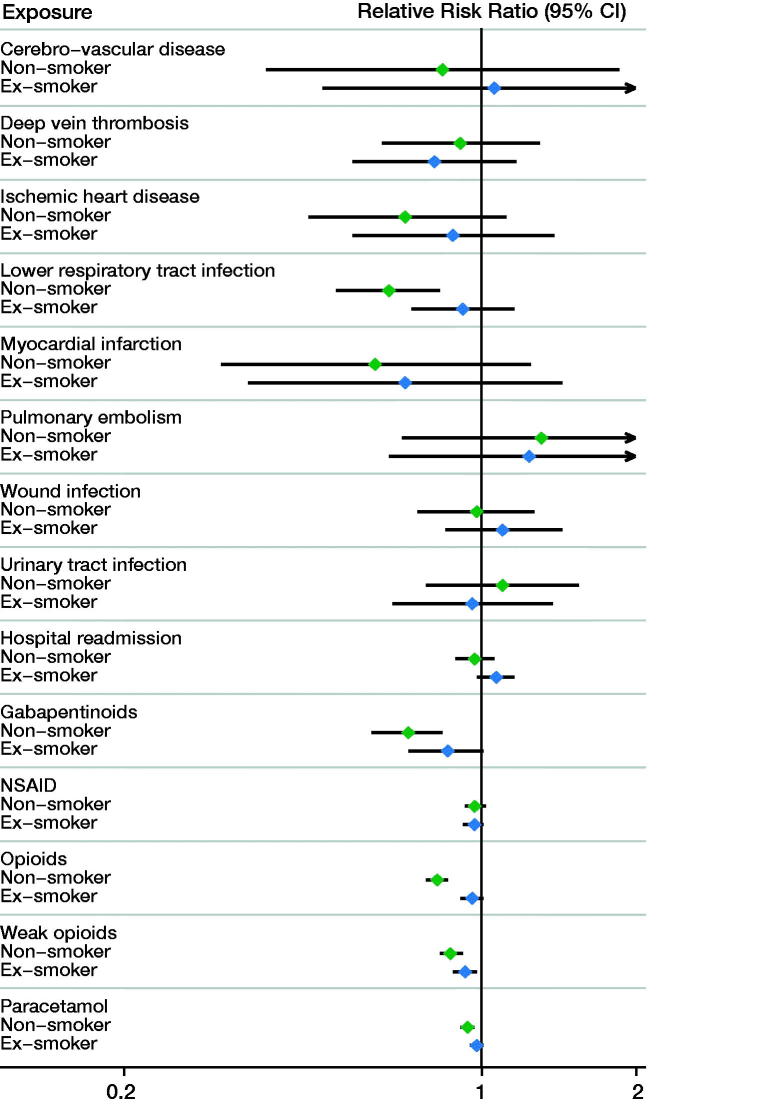
Forest plot for complications and medication usage following total knee arthroplasty by smoking status. The respective relative risk ratios and 95% confidence intervals are provided in Appendix 3.

Following THA, smokers had an increased risk of myocardial infarction and LRTI compared with both non-smokers and ex-smokers (Figure 1; Appendix 2 and 3). Smokers had a similar risk of wound infection and thromboembolic events compared with non-smokers and ex-smokers following THA as well as TKA. Following TKA, only LRTI was more commonly observed in smokers compared with non-smokers (RRR = 0.66, CI 0.52–0.83) (Figure 2; Appendix 3 and 4).

### Medication usage

Within 1 year of THA, smokers had increased use of opioids compared with non-smokers (RRR = 0.65; CI 0.61–0.69) and ex-smokers (RRR = 0.90; CI 0.84–0.95) (Figure 1; Appendix 2 and 3). Smokers also had increased use of weak opioids, paracetamol, and gabapentinoids compared with both non-smokers and ex-smokers following THA. Similar patterns of increased analgesia use in smokers were observed following TKA (Figure 2; Appendix 3 and 4).

### Readmission

The risk of hospital readmission in smokers following THA was higher compared with non-smokers, but not compared with ex-smokers (Figure 1). The risk of hospital readmission was not affected by smoking status following TKA (Figure 2).

### Revision surgery

The risk of revision up to 20 years following THA was similar in smokers compared with non-smokers (hazard ratio (HR) = 1.1; CI 0.88–1.5) and ex-smokers (HR = 1.1; CI 0.84–1.5). The risk of revision following TKA was similar in smokers compared with non-smokers (HR = 1.2; CI 0.90–1.6) and ex-smokers (HR 1.1; CI = 0.78–1.4).

### Mortality

**Figure 3.**
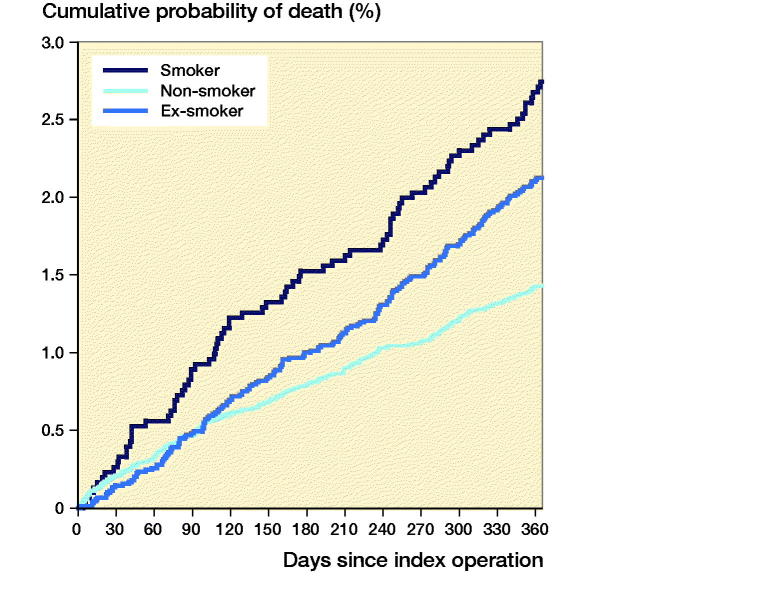
Cumulative probability of mortality up to 1 year following total hip arthroplasty.

**Figure 4.**
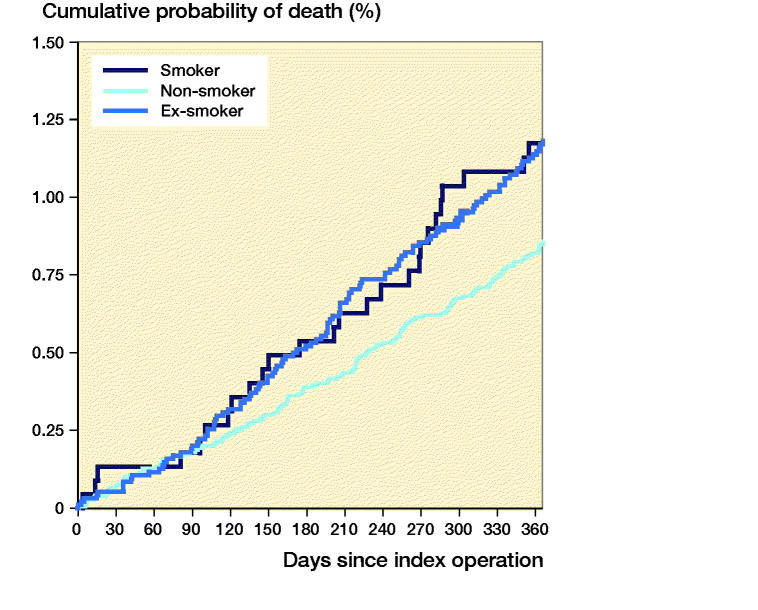
Cumulative probability of mortality up to 1 year following total knee arthroplasty.

Following THA, 1-year mortality rates were higher in smokers compared with non-smokers (HR = 0.37, CI 0.29–0.49) and ex-smokers (HR = 0.53, CI 0.40–0.70) (Figure 3). Following TKA, 1-year mortality rates were higher in smokers compared with non-smokers only (HR = 0.52, CI 0.34–0.81), but not compared with ex-smokers (HR = 0.71, CI 0.46–1.1) (Figure 4).

### Patient-reported outcome measures

**Figure 5.**
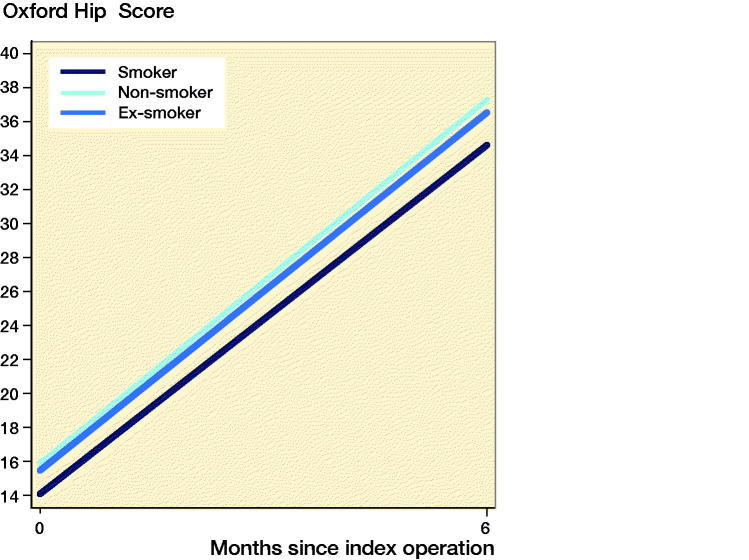
Estimation of the mean predicted preoperative (0 months) and postoperative (6 months) Oxford Hip Score by smoking status for patients receiving total hip arthroplasty.

**Figure 6.**
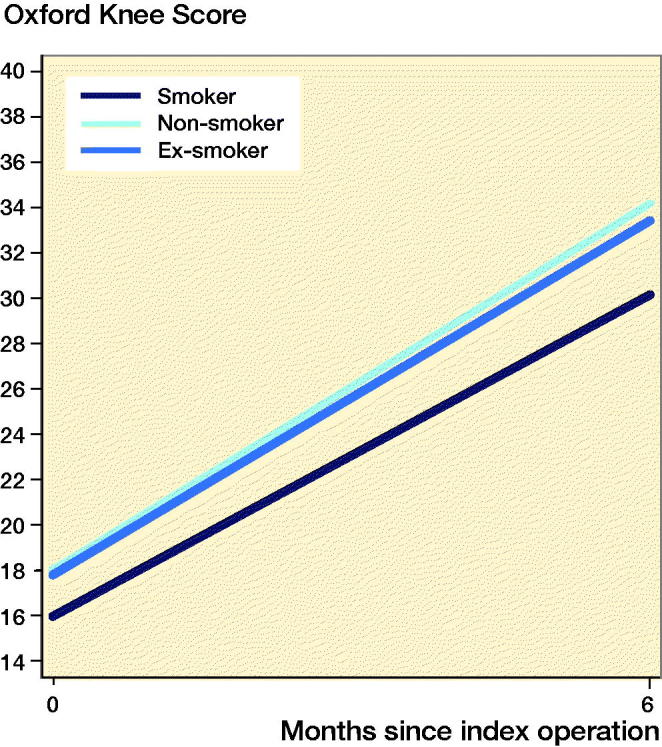
Estimation of the mean predicted preoperative (0 months) and postoperative (6 months) Oxford Knee Score by smoking status for patients receiving total knee arthroplasty.

PROMs were available for 10,009 (8.6%) patients. Smokers had improvement in PROMs compared with the preoperative scores (Figures 5 and 6, and Appendix 5). Smokers undergoing THA and TKA had lower postoperative PROMs compared with non-smokers and ex-smokers; however, these differences were not clinically meaningful. Following THA, smokers had lower postoperative OHSs compared with non-smokers (mean 2.5 points; CI 1.5–3.5) and ex-smokers (mean 1.8 points; CI 0.79–2.9). Following TKA, smokers had lower postoperative OKSs compared with non-smokers (mean 3.2 points; CI 2.0–4.5) and ex-smokers (mean 2.9 points; CI 1.7–4.2).

## Discussion

Smoking was associated with an increased risk of medical complications, increased analgesia usage, and higher mortality following arthroplasty.

The increased risk of LRTI (Bohl et al. 2016) and cardiovascular complications (Ockene and Miller 1997, Hunt et al. 2017) in smokers undergoing arthroplasty in this study was similar to previous observations. However, there was no increased risk of cardiovascular complications following TKA in smokers compared with ex-smokers and non-smokers, which could represent selection bias for undergoing these procedures. Contrary to some studies (Duchman et al. 2015, Bohl et al. 2016, Sahota et al. 2018), we found no increase in wound infections in smokers following arthroplasty, and the risks of venous thromboembolism were similar between groups. The differences in cohort size and study design may explain some of these findings, including our separate analyses for THA and TKA, and differences in follow-up period. Previous studies have assessed outcomes at 30 days (Duchman et al. 2015, Sahota et al. 2018) despite needing at least 90 days to report postoperative infections (Centers for Disease Control 2018). Arthroplasty patients who smoked had higher 1-year mortality rates compared with non-smokers and ex-smokers (the latter for THA only), with similar observations reported previously (Singh et al. 2011, Clement et al. 2012). However, it is recognized that mortality rates for smokers in the general population are known to be 2 to 3 times higher compared with non-smokers (Thun et al. 2013, Carter et al. 2015).

Increased opioid usage in smokers following arthroplasty has been reported (Kim et al. 2017, Cryar et al. 2018). Within 1 year of arthroplasty we observed increased use of paracetamol, non-steroidal anti-inflammatory drugs, weak opioids (including codeine), strong opioids (including morphine), and gabapentinoids in smokers compared with non-smokers. This is concerning given the current worldwide opioid epidemic (Brat et al. 2018) coupled with the projected increase in arthroplasty (Culliford et al. 2015). This poses significant public health risks including the development of opioid dependence and opioid-related deaths (Brat et al. 2018). All clinicians must therefore remain cognizant of the increased analgesic needs of smokers following arthroplasty and use non-opioid medications when possible.

Smokers had a similar risk of long-term revision (at up to 20 years postoperatively) compared with non-smokers and ex-smokers. Other studies observing an increased revision risk have focused on short-term outcomes (Duchman et al. 2015, Teng et al. 2015). Furthermore, smokers obtained clinically meaningful PROM improvement following arthroplasty, with postoperative PROMs comparable with non-smokers and ex-smokers. Other smaller studies have suggested that smoking does not adversely influence postoperative PROMs (Fisher et al. 2007, Khan et al. 2009). Our findings therefore suggest that arthroplasty is clinically effective in smokers, which is important to recognize given the increasing pressures from some healthcare providers to deny arthroplasty to patients who continue to smoke (RCS 2016, BOA 2017, Iacobucci 2018a).

Most adverse outcomes, namely complications and mortality, in smokers (versus non-smokers) were not seen in ex-smokers. This suggests that stopping smoking prior to arthroplasty may reduce postoperative risks. Fewer studies have assessed the effect of cessation of smoking (ex-smoking) on outcomes following arthroplasty (Singh 2011). These previous studies generally suggest that ex-smoking can still be associated with complications and mortality following arthroplasty. However, this variance could again relate to methodological differences between our study and previous work.

The question of whether or not smokers should quit before arthroplasty raises an important ethical dilemma. On one hand there is a proven and clinically effective operation available that can substantially reduce the pain and disability associated with hip and knee arthritis. On the other hand, by proceeding with elective arthroplasty in current smokers there are increased risks related to postoperative medical complications, mortality, and analgesia usage, which are all arguably avoidable.

An early randomized controlled trial reported fewer postoperative complications in smokers who either quit or reduced their smoking by 50% prior to THA and TKA compared with those continuing to smoke (Moller et al. 2002). Subsequently strong evidence has been published regarding the benefits of various smoking cessation methods before surgery (Myers et al. 2011, Thomsen et al. 2014). The latest Cochrane review, which included 13 trials assessing smoking cessation, concluded that preoperative smoking interventions, which provide behavioral support and offer nicotine replacement therapy, can increase cessation in the short term and may reduce postoperative morbidity (Thomsen et al. 2014). Although the optimal preoperative intensity remains unknown, the authors suggested that interventions beginning 4 to 8 weeks before surgery, including weekly counselling and nicotine replacement therapy, were more likely to have an impact on complications and on long–term smoking cessation.

On the basis of our data and the existing literature we recommend that healthcare professionals actively promote smoking cessation preoperatively in patients eligible for arthroplasty, as this will prevent the increased surgical risk associated with smoking and ultimately will improve patient safety. Preoperative smoking cessation also has the advantage of promoting long-term smoking abstinence (Rigotti et al. 2008, Thomsen et al. 2009). However, it is somewhat concerning that smoking cessation funding is currently being substantially reduced or removed altogether in some areas of England (Iacobucci 2018b), which will undoubtedly influence who has access to this evidence-based support and the quality of it. Eligible arthroplasty patients who are unable to quit smoking despite undergoing cessation therapy will also continue to pose a dilemma. Although there is no clear evidence as to what to do in these circumstances, it is advised that the surgeon reconsiders the indication for surgery and balances the benefits and risks together with the patient, anesthesiologist, and other relevant healthcare professionals.

### Strengths and limitations

This is by far the largest study assessing the effect of smoking on outcomes following arthroplasty. We suspect the findings are generalizable to many Western populations. Contrary to other studies, we have importantly considered outcomes in ex-smokers, subdivided the cohort into THA and TKA, and explored short-term and long-term postoperative outcomes, including PROMs, implant and patient survival, and medication usage. Therefore our findings provide the most comprehensive picture of the outcomes patients will achieve following arthroplasty based on smoking status, and they provide useful information for healthcare professionals when counselling patients regarding the relative risks associated with each arthroplasty procedure.

Using observational data means we cannot infer causality. Although the validity of clinical diagnoses in CPRD is high (Herrett et al. 2010), it is possible that some inaccuracies in coding exist within the dataset analyzed. We acknowledge that a limitation of using routinely available data from primary care is that information on smoking status is captured broadly in only 3 categories. Also, more detailed information on this exposure (cigarettes per day, tar and tobacco content etc.) was not available. Thus it was not possible to analyze any potential dose–response relationship related to the outcomes. Given the length of time since stopping smoking was not known, by categorizing the short-term quitters together with long-term quitters it is possible this could increase the complication rate for the total ex-smoking group, thereby reducing a difference between the ex-smoking and smoking groups. We also recognize that former smoking may be under-recorded in CPRD, which may influence the findings in this particular group (Booth et al. 2013).

Some variables had missing data, which may have affected the findings. Most incomplete variables only had a small proportion of missing data. However, the later introduction of PROMs into HES (and availability of only 6-month post-surgery PROMs) means that much fewer patients had PROMs available. Thus care should be taken when interpreting results relating to PROMs. Some outcome measures, such as revision, can be underestimated (Sabah et al. 2016), but there is no reason to suspect any underestimation would differ between the exposure groups. Although we identified differences in all-cause mortality between smoking groups, data were not available on cause-specific mortality; therefore we cannot comment on the causes of death and whether they were smoking related. Furthermore, given how the CPRD population was selected, our findings may not apply to other populations worldwide with different patient characteristics and/or healthcare practices.

### Conclusions

This large observational cohort study demonstrated that smokers gain benefit from arthroplasty, with clinically meaningful PROM improvement and no increased revision risk. However, smoking was associated with more medical complications, analgesia usage, and death following arthroplasty. Most adverse outcomes were reduced in ex-smokers compared with smokers, suggesting that preoperative smoking cessation may improve outcomes following arthroplasty and thus should be encouraged by healthcare professionals. 

### Supplementary data

Appendices 1–5 are available as supplementary data in the online version of this article, http://dx.doi.org/10.1080/ 17453674.2019.1649510

## Supplementary Material

Supplemental Material
