# Supplementary material for: The effect of smoking on outcomes following primary total hip and knee arthroplasty: a population-based cohort study of 117,024 patients
Source: Acta Orthop. 2019 Aug 2;90(6):559–67. doi: 10.1080/17453674.2019.1649510 (PMC6844375; doi:10.1080/17453674.2019.1649510)
Supplement: Supplemental Material [file IORT_A_1649510_SM1781.pdf]

Supplementary data

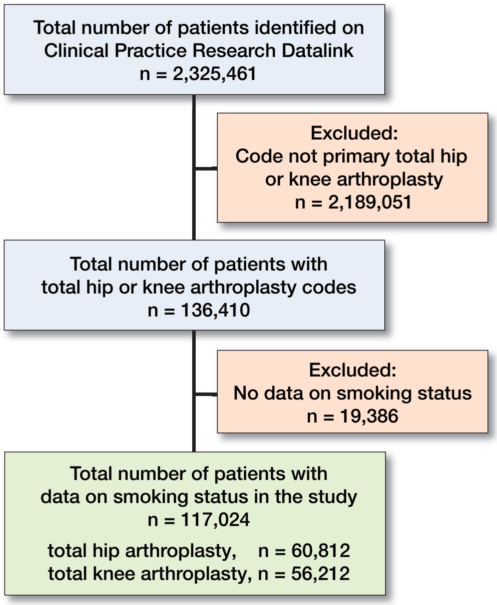

Appendix 1. Study selection criteria

Appendix 2. Outcomes following total hip arthroplasty. Values are frequency (%) unless otherwise stated

Outcomes	Smoker	Non-smoker	Ex-smoker
Complications			
Any (≥ 1 complication)	826 (11)	3,260 (10)	2,161 (11)
Cerebrovascular disease	41 (0.5)	163 (0.5)	75 (0.4)
Deep vein thrombosis	120 (1.6)	571 (1.7)	290 (1.5)
Ischemic heart disease	67 (0.9)	290 (0.8)	206 (1.1)
LRTI	353 (4.7)	892 (2.6)	817 (4.3)
Myocardial infraction	38 (0.5)	111 (0.3)	84 (0.4)
Pulmonary embolism	51 (0.7)	270 (0.8)	153 (0.8)
Postop. wound infection	141 (1.9)	593 (1.7)	388 (2.0)
Urinary tract infection	127 (1.7)	706 (2.1)	415 (2.2)
Readmission to the hospital	799 (11)	3,370 (9.9)	2,264 (12)
Medications			
Gabapentinoids	469 (6.2)	1,435 (4.2)	946 (5.0)
NSAID	3,134 (42)	12,040 (35)	6,963 (37)
Opioids	2,805 (37)	7,858 (23)	5,985 (32)
Weak opioids	2,952 (39)	11,301 (33)	6,612 (35)
Paracetamol	4,473 (59)	18,677 (55)	11,232 (59)
Mortality within			
1 year after THA	189 (2.5)	528 (1.5)	379 (2.0)
Postoperative Oxford Hip Score			
median (IQR)	41 (32–45)	43 (36–46)	42 (35–46)

LRTI: Lower respiratory tract infection

Appendix 5. Estimation of the mean predicted preoperative (0 months) and postoperative (6 months) Oxford Hip and Knee Scores by smoking status with 95% confidence intervals (CI)

Score	Time of score	Predicted score mean (CI)
Oxford Hip Score		
Smoker	Preoperative	14 (13–15)
	6 months postoperative	35 (33–36)
Non-smoker	Preoperative	16 (15–17)
	6 months postoperative	37 (36–38)
Ex-smoker	Preoperative	16 (15–16)
	6 months postoperative	37 (36–38)
Oxford Knee Score		
Smoker	Preoperative	16 (14–18)
	6 months postoperative	30 (28–32)
Non-smoker	Preoperative	18 (17–20)
	6 months postoperative	34 (33–36)
Ex-smoker	Preoperative	18 (16–19)
	6 months postoperative	33 (32–35)

Appendix 4. Outcomes following total knee arthroplasty. Values are frequency (%) unless otherwise stated

Outcomes	Smoker	Non-smoker	Ex-smoker
Complications			
Any (≥ 1 complication)	562 (11)	3,287 (10)	2,209 (12)
Cerebrovascular disease	20 (0.4)	110 (0.3)	82 (0.4)
Deep vein thrombosis	78 (1.5)	502 (1.6)	288 (1.5)
Ischemic heart disease	45 (0.9)	245 (0.8)	209 (1.1)
LRTI	214 (4.2)	874 (2.7)	744 (3.9)
Myocardial infraction	19 (0.4)	87 (0.3)	71 (0.4)
Pulmonary embolism	33 (0.6)	265 (0.8)	165 (0.9)
Postop. wound infection	149 (2.9)	901 (2.8)	573 (3.0)
Urinary tract infection	75 (1.5)	669 (2.1)	309 (1.6)
Readmission to the hospital	671 (13)	4,150 (13)	2,816 (15)
Medications			
Gabapentinoids	377 (7.4)	1,652 (5.2)	1,075 (5.6)
NSAID	2,633 (52)	14,741 (46)	8,870 (46)
Opioids	2,342 (46)	11,291 (35)	7,799 (41)
Weak opioids	2,348 (46)	13,323 (42)	8,037 (42)
Paracetamol			
Mortality within			
1 year after TKA	55 (1.1)	277 (0.9)	217 (1.1)
Postoperative Oxford Knee Score			
median (IQR)	35 (22–41)	38 (30–43)	37 (30–43)

LRTI: Lower respiratory tract infection

Appendix 3. Univariable (crude) and multivariable (adjusted) regression models for each outcome of interest. The respective regression ratios are shown with 95% confidence intervals in brackets

	THA		TKA	
Outcome	univariable	multivariable	univariable	multivariable
Cerebrovascular disease				
Non-smoker	0.87 (0.50–1.53)	0.56 (0.31–1.04)	1.19 (0.54–2.60)	0.84 (0.38–1.86)
Ex-smoker	0.75 (0.41–1.37)	0.45 (0.23–0.85)	1.73 (0.79–3.82)	1.06 (0.49–2.31)
Deep vein thrombosis				
Non-smoker	0.98 (0.73–1.32)	0.95 (0.70–1.29)	0.99 (0.70–1.40)	0.91 (0.64–1.30)
Ex-smoker	0.92 (0.67–1.26)	0.92 (0.66–1.27)	0.90 (0.63–1.29)	0.81 (0.56–1.17)
Ischemic heart disease				
Non-smoker	0.75 (0.51–1.09)	0.60 (0.41–0.88)	0.76 (0.49–1.19)	0.71 (0.46–1.12)
Ex-smoker	1.02 (0.69–1.51)	0.80 (0.53–1.21)	1.05 (0.67–1.64)	0.88 (0.56–1.39)
Lower respiratory tract infection				
Non-smoker	0.59 (0.49–0.71)	0.53 (0.44–0.64)	0.70 (0.56–0.87)	0.66 (0.52–0.83)
Ex-smoker	0.94 (0.78–1.13)	0.82 (0.67–0.99)	0.98 (0.78–1.22)	0.92 (0.73–1.16)
Myocardial infarction				
Non-smoker	0.55 (0.32–0.96)	0.36 (0.21–0.63)	0.68 (0.34–1.34)	0.62 (0.31–1.25)
Ex-smoker	0.72 (0.40–1.28)	0.45 (0.25–0.81)	0.98 (0.49–1.95)	0.71 (0.35–1.44)
Pulmonary embolism				
Non-smoker	0.92 (0.61–1.41)	0.85 (0.56–1.31)	1.59 (0.86–2.95)	1.31 (0.70–2.44)
Ex-smoker	0.88 (0.56–1.37)	0.80 (0.51–1.26)	1.45 (0.77–2.74)	1.24 (0.66–2.34)
Wound infection				
Non-smoker	1.07 (0.81–1.43)	1.06 (0.80–1.42)	0.96 (0.75–1.24)	0.98 (0.75–1.27)
Ex-smoker	1.22 (0.91–1.64)	1.19 (0.88–1.60)	1.03 (0.80–1.34)	1.10 (0.85–1.44)
Urinary tract infection				
Non-smoker	1.15 (0.87–1.51)	0.87 (0.66–1.16)	1.50 (1.07–2.11)	1.10 (0.78–1.55)
Ex-smoker	1.28 (0.96–1.70)	1.09 (0.82–1.47)	1.11 (0.78–1.59)	0.96 (0.67–1.38)
Readmission				
Non-smoker	0.85 (0.79–0.92)	0.83 (0.76–0.90)	0.96 (0.88–1.04)	0.97 (0.89–1.06)
Ex-smoker	1.03 (0.94–1.12)	0.96 (0.88–1.05)	1.08 (0.99–1.18)	1.07 (0.98–1.16)
Gabapentinoids				
Non-smoker	0.68 (0.58–0.80)	0.73 (0.62–0.86)	0.70 (0.60–0.82)	0.72 (0.61–0.84)
Ex-smoker	0.80 (0.68–0.95)	0.83 (0.70–0.99)	0.80 (0.68–0.94)	0.86 (0.72–1.01)
NSAIDs				
Non-smoker	0.88 (0.83–0.92)	0.94 (0.89–0.99)	0.90 (0.86–0.94)	0.97 (0.93–1.02)
Ex-smoker	0.89 (0.84–0.94)	0.97 (0.92–1.02)	0.88 (0.84–0.92)	0.97 (0.92–1.01)
Opioids				
Non-smoker	0.61 (0.57–0.64)	0.65 (0.61–0.69)	0.76 (0.72–0.80)	0.82 (0.78–0.86)
Ex-smoker	0.88 (0.82–0.93)	0.90 (0.84–0.95)	0.88 (0.84–0.93)	0.96 (0.91–1.01)
Weak opioids				
Non-smoker	0.85 (0.81–0.90)	0.82 (0.78–0.87)	0.88 (0.84–0.93)	0.87 (0.83–0.92)
Ex-smoker	0.89 (0.85–0.95)	0.91 (0.86–0.97)	0.89 (0.85–0.94)	0.93 (0.88–0.98)
Paracetamol				
Non-smoker	0.94 (0.91–0.98)	0.87 (0.84–0.90)	0.98 (0.95–1.01)	0.94 (0.91–0.97)
Ex-smoker	1.03 (0.99–1.07)	0.96 (0.93–1.00)	0.99 (0.96–1.03)	0.98 (0.95–1.01)
Mortality				
Non-smoker	0.51 (0.40–0.66)	0.37 (0.29–0.49)	0.73 (0.48–1.11)	0.52 (0.34–0.81)
Ex-smoker	0.77 (0.60–1.00)	0.53 (0.40–0.70)	1.00 (0.65–1.53)	0.71 (0.46–1.10)
Revision surgery				
Non-smoker	0.95 (0.74–1.21)	1.13 (0.88–1.47)	0.86 (0.66–1.13)	1.19 (0.90–1.58)
Ex-smoker	0.96 (0.73–1.25)	1.10 (0.84–1.46)	0.78 (0.58–1.04)	1.06 (0.78–1.42)

THA = total hip arthroplasty; TKA = total knee arthroplasty.
Smoking group is the reference group in all cases.
